# Systematic Review of Hansen Disease Attributed to *Mycobacterium lepromatosis*

**DOI:** 10.3201/eid2907.230024

**Published:** 2023-07

**Authors:** Simon M. Collin, Amanda Lima, Stéfano Heringer, Vinícius Sanders, Hugo Aborghetti Pessotti, Patrícia Deps

**Affiliations:** Universidade Federal do Espírito Santo, Vitória, Espírito Santo, Brazil

**Keywords:** *Mycobacterium lepromatosis*, Hansen disease, leprosy, mycobacteria, bacteria, zoonoses, Brazil, tuberculosis and other mycobacteria, Mexico, United States

## Abstract

In 2008, bacilli from 2 Hansen disease (leprosy) cases were identified as a new species, *Mycobacterium lepromatosis*. We conducted a systematic review of studies investigating *M. lepromatosis* as a cause of HD. Twenty-one case reports described 27 patients with PCR–confirmed *M. lepromatosis* infection (6 dual *M. leprae*/*M. lepromatosis*): 10 case-patients in the United States (7 originally from Mexico), 6 in Mexico, 3 in the Dominican Republic, 2 each in Singapore and Myanmar, and 1 each in Indonesia, Paraguay, Cuba, and Canada. Twelve specimen surveys reported 1,098 PCR–positive findings from 1,428 specimens, including *M. lepromatosis* in 44.9% (133/296) from Mexico, 3.8% (5/133) in Colombia, 12.5% (10/80) in Brazil, and 0.9% (2/224) from the Asia-Pacific region. Biases toward investigating *M. lepromatosis* as an agent in cases of diffuse lepromatous leprosy or from Mesoamerica precluded conclusions about clinicopathologic manifestations and geographic distribution. Current multidrug treatments seem effective for this infection.

Since the pioneering work of Gerhard Armauer Hansen in the late 19th Century, Hansen disease (HD; also known as leprosy) has been attributed to *Mycobacterium leprae*. In 2008, bacilli from 2 cases of HD manifesting as diffuse lepromatous leprosy (DLL; also known as Lucio’s leprosy or diffuse leprosy of Lucio and Latapi), with signs of Lucio’s phenomenon (LP, erythema necroticans), were identified as a second causal agent of HD, *M. lepromatosis* (*1*). The *M. lepromatosis* genome has been sequenced and its evolution and genomic features in relation to *M. leprae* described elsewhere (*2*–*5*). Analyses indicated a most recent common ancestor ≈13.9 million years ago and a 9% overall difference in nucleotide sequence identity (7% in protein-coding genes, 18% in pseudogenes), differentiating *M. lepromatosis* as a separate species from *M. leprae* (*3*,*4*). Functional similarities, such as conservation of genes encoding for laminin binding and phenolic glycolipid 1 adhesin systems involved in Schwann cell invasion, outweigh differences, such as the presence of the hemN gene only in *M. lepromatosis* (*3*,*4*). Whether the 2 species differ systematically in clinicopathologic manifestations in humans has not yet been established, but validated real-time quantitative PCR assays based on unique repetitive elements in *M. lepromatosis* and *M. leprae* are now available (*6*). 

DLL is a severe form of HD at the lepromatous pole of the spectrum characterized by an ineffective cellular immune response and high multibacillary load (*7*). Patients with DLL manifest diffuse nonnodular lesions and can develop LP, a severe reactional state in which recurrent crops of large and sharply demarcated ischemic or necrotic skin develop; the lesions often becoming ulcerated or even generalized, particularly on the legs, leading to secondary infection and, in some cases, fatal sepsis (*8*). DLL represents a higher proportion of HD cases in Mexico and the Caribbean than elsewhere, and studies reporting *M. lepromatosis* have tended to describe patients who originate from the region with that form of HD (*9*). However, dual *M. leprae*/*M. lepromatosis* and *M. lepromatosis*–only infections have also been reported beyond the Americas, principally in Asia. 

Worldwide occurrences and clinical characteristics of HD attributed to *M. lepromatosis* infection since the species was identified have not been systematically reviewed. There is a clinical and scientific imperative to clarify the contribution of *M. lepromatosis* to a disease that greatly affects patient and public health. We report the results of a systematic review of reported HD cases with PCR–confirmed *M. lepromatosis* infection and data from surveys of archived PCR–tested specimens from persons affected by HD. 

## Methods 

### Review Protocol and Searches

The protocol for this systematic review was defined in advance and registered with PROSPERO, an international prospective register of systematic reviews (CRD42021239268). Database searches were performed on October 4, 2022 (Appendix). We imposed no date, language, or publication type restrictions. We manually searched bibliographies of all included studies. 

### Screening, Inclusion/Exclusion, and Quality Assessment 

We conducted initial screening by title and abstract. We included references if a primary research study or clinical case report reported human infection with *M. lepromatosis* investigated using laboratory testing of current or archived specimens, irrespective of whether those specimens were positive for *M. lepromatosis*. We excluded animal studies and studies from before 2008, predating identification of *M. lepromatosis.* We excluded reviews and opinion pieces after manually checking bibliographies. Pairs of reviewers in parallel performed qualitative assessments to rate the methodologic quality of each included study as good, fair, or poor (Appendix). Reviewers used the Joanna Briggs Institute Critical Appraisal Tool for Case Reports (https://jbi.global/critical-appraisal-tools) and, for specimen surveys, a 9-item quality assessment tool adapted from the National Institutes of Health’s Quality Assessment Tool for Observational Cohort and Cross-Sectional Studies (https://www.nhlbi.nih.gov/health-topics/study-quality-assessment-tools) (*10*). 

### Data Extraction and Analysis

Pairs of reviewers in parallel transferred extracted data into predefined templates. Data extracted from case reports were date and geographic location of testing, patient demographics, medical history, diagnostic methods and findings, treatment, and outcome. Data extracted from surveys were case information, test methods, source and type of specimens, and how many specimens provided DNA and tested positive for *M. lepromatosis*, *M. leprae*, or both. 

## Results

**Figure 1:**
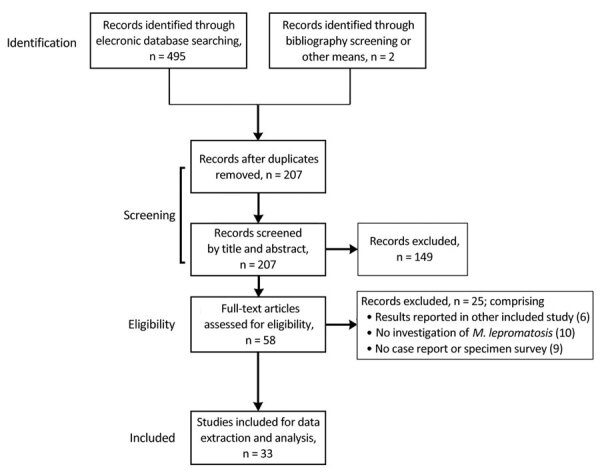
Flow diagram for studies included in literature review of Hansen disease attributed to *Mycobacterium lepromatosis*.

Database searches identified 495 references (Figure 1; Appendix). We identified no additional studies through bibliographic screening, but for completeness, we did include 2 case reports published after our database searches (*9*,*11*). After de-duplication and screening by title and abstract, we retained 58 studies, all published in peer-reviewed journals, for full text review; we extracted data from 33 (21 case reports, 12 specimen surveys). We excluded 1 because it was a retrospective review of 9 cases (*12*) that reported only 1 *M. lepromatosis* case that was also described in another source (*13*). Similarly, we excluded a review of cases among refugees and migrants in Italy during 2009–2018 (*14*) that reported PCR testing of 24 cases, 16 positive for *M. leprae* and 1 for *M. lepromatosis*, because the *M. lepromatosis* case was described in more detail in a case report (*15*). 

Among the 21 case report studies, 14 studies described just 1 case, 6 described 2, and 1 described 6, yielding 32 PCR–positive cases: 21 *M. lepromatosis*–only, 5 *M. leprae–*only, and 6 dual infections (Table 1; Appendix). Of patients with *M. lepromatosis*, 10/27 resided in the United States (7 originally from Mexico), 6 in Mexico, 3 in the Dominican Republic, 2 each in Singapore and Myanmar, and 1 each in Indonesia, Paraguay, Cuba, and Canada. One study from Mexico reported 4 family cases, but only 2 were PCR–confirmed to be *M. lepromatosis* (*9*). Twenty-two cases occurred in the Americas and 5 in Asia; Mexico was the country of origin or residence for 13/27 case-patients. One source mentioned 2 case-patients from Costa Rica living in the United States but provided no details (*23*). Median age of case-patients was 41 years (range 21–86 years) and 63.0% (17/27) were male. 

**Table 1:** Case reports and case series investigating *Mycobacterium lepromatosis* as a cause of Hansen disease (chronological by year of publication)*

Ref.	YOP	Patient residence (origin)	Patient age, y/sex	Case description	Specimen source	PCR-confirmed infection	Remarks
(*1*)	2008	USA (Mexico)	53/M	DLL + LP	Autopsy†	*M. lepromatosis*	Fatal. *M. lepromatosis* strain FJ924.
		USA (Mexico)	M/31	DLL + LP	Archive	*M. lepromatosis*	Fatal. Archived lymph node tissue (2002).
(*16*)	2011	Mexico	F/86	DLL + LP	Archive	*M. lepromatosis*	Died at 3 mo. Strain Mx1-22 (100% identical with FJ924).
(*17*)	2012	Singapore	M/61	DLL	Archive	Dual	Fatal. Archived skin biopsy tissue (1999).
		Singapore	M/72	DLL	Archive	Dual	Fatal. Archived skin biopsy tissue (1999).
(*18*)	2012	Canada	M/72	Leprosy-like‡	Current patient§	*M. lepromatosis*	Died (lung cancer) at 5 mo. Some travel to Florida, no other risk factors.
(*19*)	2013	USA (Mexico)	M/32	LL + ENL	Archive	*M. lepromatosis*	Archived lymph node tissue (2005).
		USA (Mexico)	F/50	DLL + LP	Archive	*M. lepromatosis*	Archived lymph node tissue (1963).
(*20*)	2015	Mexico	F/43	DLL + ENL	Current patient	*M. lepromatosis*	Armadillo meat eaten in community but not by patient.
(*22*)	2016	Colombia	F/37	HIV+, LL + LP	Archive	*M. leprae*	LP possible IRIS because of ART.
(*23*)	2016	USA (Mexico)	M/25	BL + T1R >ENL¶	Archive	*M. lepromatosis*	Patient reported handling and eating armadillo in Mexico. Syphilis (borderline positive ANA).
		USA (Mexico)	F/41	BL + ENL	Current patient	*M. lepromatosis*	Manifested 2012, sibling of 25 y M (2007 case), cohabited with brother for 12 mo after arrival in USA (2003).
(*24*)	2016	Mexico	M/49	DLL + ENL >LP¶	Current patient	*M. lepromatosis*	None
(*25*)	2017	USA (Mexico)	M/31	DLL + ENL	Current patient	*M. lepromatosis*	Patient reported hunting and eating armadillo in Mexico.
(*13*)	2017	USA	M/59	LL	Current patient#	*M. lepromatosis*	Rheumatoid arthritis for 2 y (prednisone + methotrexate).
(*26*)	2018	Myanmar	M/68	LL	Current patient	*M. lepromatosis*	Patient had HD 20 y previously (treated with dapsone).
		Myanmar	M/24	LL + ENL	Current patient	*M. lepromatosis*	BI 5+ at 24 mo, resistance suspected but no DRDR mutations.
(*21*)	2019	Indonesia	F/41	DLL + LP	Current patient	Dual	HD 28 y previous (treatment not reported).
(*27*)	2020	Paraguay	F/21	LL + ENL >LP¶	Current patient	Dual	Vasculitis had been suspected related to drug use.
(*28*)	2020	USA (Nepal)	F/43	BB + T1R >LP¶	Current patient	*M. leprae*	None
(*15*)	2020	Italy (Cuba)	F/42	DLL + LP	Current patient**	*M. lepromatosis*	Necrotic cutaneous vasculitis 4 y previous, partial resolution with corticosteroids.
(*29*)	2020	USA	M/58	LL + ENL	Current patient	*M. lepromatosis*	Patient had 12 y history of poor wound healing refractory to immunosuppressive treatment, connective tissue disease (scleroderma) and bilateral acro-osteolysis with amputated digits.
(*30*)	2021	Mexico	F/32	DLL + ENL	Current patient	*M. lepromatosis*	None
(*31*)	2022	DR	M/40	LL	Current patient	*M. lepromatosis*	None
		DR	M/35	LL	Current patient	Dual	None
		DR	F/26	LL	Current patient	Dual	Detected as contact of a family case-patient, pregnant at time of diagnosis, MDT initiated after delivery.
		DR	M/48	BL	Current patient	*M. leprae*	Detected as contact of a family case-patient
		DR (Haiti)	M/27	LL	Current patient	*M. leprae*	Detected as contact of a family case-patient
		DR	F/39	LL	Current patient	*M. leprae*	None
(*9*)	2022	Mexico	M/27	DLL + LP	Current patient††	*M. lepromatosis*	ENL at 3 y treated with thalidomide. No regrowth of eyelashes or eyebrows. Alcoholism, drug, and solvent abuse.
		Mexico	F/49	DLL	Current patient	*M. lepromatosis*	Follow up to 2021; no regrowth of eyelashes or eyebrows.
(*11*)	2022	USA	M/51	DLL + LP	Current patient	*M. lepromatosis*	Acute kidney injury/glomerulonephritis

*ART, antiretroviral therapy; BB, midborderline (borderline borderline); BI, bacillary index; BL, borderline lepromatous; DLL, diffuse lepromatous; DR, Dominican Republic; ENL, erythema nodosum leprosum (type 2 reaction); IRIS, immune reconstitution inflammatory syndrome; LL, lepromatous; LP, Lucio’s phenomenon (erythema necroticans); MDT, multidrug therapy; ref., reference; T1R, type 1 reaction; YOP, year of publication. †First case identified as *M. lepromatosis* based on the 16S rRNA (strain FJ924) unique 19bp sequence TAATACTTAAACCTATTAA that compared poorly with the corresponding unique *M. leprae* 16bp sequence AAAAAATC—TTTTTTAG. ‡No bacilli detected in nerves. §Based on report by Han et al. (2008), 16S rRNA sequencing was repeated from archived specimens and found to match 100% with *M. lepromatosis*¶Right-arrow (>) indicates clinical progression or later development of reactional states. #One of 3 cases tested by PCR as described in a retrospective clinic review of 9 cases (the other 2 cases were *M. leprae*) (*12*). **One of 24 cases in Italy tested by PCR as described in a retrospective review of 55 cases (16 cases were *M. leprae.* 7 were PCR–negative) (*23*). ††Index case believed infected by grandfather (LL in 1993, age 60 y, died 2004); infected mother (PCR-confirmed *M. lepromatosis*), and sibling (BL in 2014, age 25 y, MDT 12 mo, cured and followed up to 2021).

We assessed 13/21 studies as good and 8/21 as fair quality (Appendix). Eight studies did not provide detailed PCR methods (*11*,*15*,*22*,*24*–*26*,*28*,*29*), but 7 of these referred to laboratories (National Hansen’s Disease Programme; US Centers for Disease Control and Prevention; Ecole Polytechnique Fédérale de Lausanne; Japan Leprosy Research Centre) or involved authors with documented experience in *M. lepromatosis* diagnostic methods (*11*,*22*,*24*–*26*,*28*,*29*). 

The case-patient from the study in which *M. lepromatosis* was first identified (*1*) was a patient originally from Mexico residing in the United States who had died from DLL with LP. PCR sequencing of the ≈1,500 bp 16S rRNA gene in acid-fast bacilli from frozen liver autopsy specimens showed that the strain, designated FJ924, matched most closely with *M. leprae* (BLAST analysis [https://blast.ncbi.nlm.nih.gov/Blast.cgi] of 16S rRNA gene, 1,475/1,506 bp, 97.9% identity) and next most closely with *M. haemophilum* (1,465/1,505 bp, 97.3%). The researchers obtained archived biopsy specimens from a second patient originally from Mexico, also with DLL and LP, who had died 5 years earlier. Gene sequences from that earlier case, including from the 16S rRNA gene, matched 100% with strain FJ924. On the basis of those findings, the researchers proposed a new species, *M. lepromatosis*, as a second causal agent of DLL, while speculating that it might also cause lepromatous (LL) and borderline lepromatous (BL) forms of HD (*1*). The researchers also obtained archived specimens from 2 fatal cases of DLL in Singapore (both case-patients died in 1999) with dual *M. lepromatosis*/*M. leprae* infection identified using a mix of species-specific and nonspecific primers matched to GenBank sequences (*17*). 

In another study from Mexico, a sample, Mx1-22, taken from an 86-year-old patient with DLL and LP had *rrs*, *rpoB*, *sigA*, and *hsp65* gene sequences identical to FJ924 (*16*). Subsequent studies used a range of species-specific primers and sequencing, 7 targeting 16S rRNA (*13*,*18*–*21*,*23*,*30*), 2 *hemN* (*27*,*31*), and 1 the LPMREP repetitive element (*9*) to confirm *M. lepromatosis* infection (Table 1). The oldest archived specimen in which *M. lepromatosis* was identified was from a US-resident patient originally from Mexico, 50 years of age, treated in Carville, Louisiana, USA, who was diagnosed with DLL with LP in 1963 (*19*). That patient, who initially sought treatment for a soft tissue sarcoma in the right lower leg, developed overt signs of DLL and LP after radiotherapy and amputation of the leg. Histopathologic review identified chronic HD lesions in the skin, vessels, and nerves surrounding the sarcoma, consistent with DLL. The patient survived to 85 years of age. 

**Figure 2:**
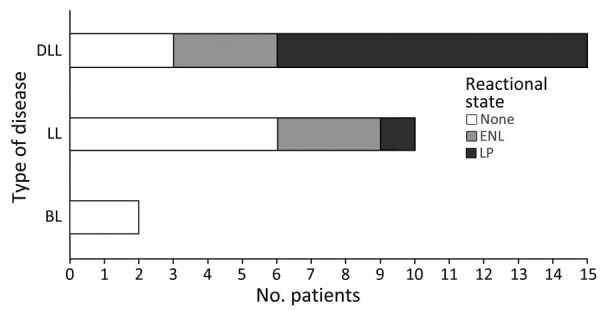
Types of Hansen disease and reactional states in reports included in literature review of PCR-confirmed cases attributed to *M. lepromatosis*. BL, borderline lepromatous leprosy; DLL, diffuse lepromatous leprosy; ENL, erythema nodosum leprosum (type 2 reaction); LL, lepromatous leprosy; LP, Lucio’s phenomenon (erythema necroticans, type 3 reaction)

**Figure 3:**
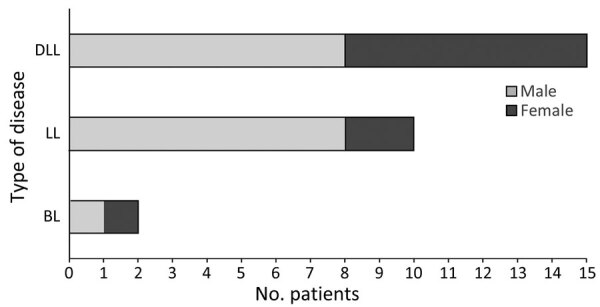
Types of Hansen disease by case-patient sex in reports included in literature review of PCR-confirmed cases attributed to *M. lepromatosis*. BL, borderline lepromatous leprosy; DLL, diffuse lepromatous leprosy; LL, lepromatous leprosy.

Of the 27 *M. lepromatosis* case-patients, 15 (55.6%) had DLL, 9 of whom also had LP; 10 had LL, 1 had LP, and 2 had BL (Figure 2). Among those cases, type 2 erythema nodosum leprosum (ENL) HD reactions were reported in 3 cases each of DLL and LL and in both BL cases. Male patients comprised 8/15 DLL and 1/2 BL case-patients but a higher proportion (8/10) of LL case-patients (Figure 3). Median time of evolution from initial symptoms to HD diagnosis was 2 years (range 8 months–12 years); 2 patients had been diagnosed with HD 20 and 28 years earlier. Two patients reported direct contact (hunting, handling, or eating) with armadillos in Mexico (*23*,*25*); a third came from a village in Mexico where armadillo meat was consumed, but the patient had not eaten it (*20*). Two patients in the United States had no known risk factors or exposures other than travel, including to Florida (*18*), worldwide travel including to Asia, the Caribbean, and the Middle East, and 2 trips to the Pacific coast of Mexico that were short but occurred consistent with a 7–8 year incubation period for HD manifestation (*13*). 

Concomitant or differential diagnoses discussed in the case reports included sarcoidosis (initially treated with steroids) (*19*), syphilis (borderline positive antinuclear antibodies, treated initially with intramuscular penicillin) (*23*), rheumatoid arthritis (treated with prednisone and methotrexate 2 years earlier) (*13*), cutaneous vasculitis (treated with azathioprine and prednisone for >5 years) (*30*), vasculitis related to drug abuse (*27*), and acute kidney injury (*11*). All cases were otherwise consistent with the clinical and histopathologic picture of DLL (*32*): insidious onset with violaceous erythema developing on the face and lower extremities (may or may not be anesthetic); myxoedema-like aspect with smooth, tense, alopecic skin, progressing to madarosis; earlobe infiltration; rhinitis; nasal septal defects; hypohidrosis; xerotic and scaly skin with ichthyosiform appearance on lower limbs; areas of hypoesthesia and hyperesthesia associated with hypopigmented, atrophic plaques; and impaired sensation in the hands and feet becoming more generalized because of progressive nerve involvement. Histologically, dense histiocytic infiltration in skin and nerves was observed, advancing to endothelial proliferation with thickening of vascular walls, leading to occlusion of small arteries, and invasion of internal organs, indicated by hepatomegaly and splenomegaly. 

All multibacillary HD case-patients were treated with multidrug therapy, typically with rifampicin, clofazimine, and dapsone (sometimes substituted with minocycline, clarithromycin, moxifloxacin, or oxfloxacin) for 12 or 24 months, plus corticosteroids (mainly prednisone), thalidomide, or both for ENL and LP. Treatment outcomes were favorable for 10/27 patients at time of reporting, although that group included 2 patients who had no regrowth of eyelashes or eyebrows at 3-year follow-up, 1 of whom also experienced ENL at 3 years (*9*). One patient whose mild neurologic deficits had resolved at 7 years was still taking thalidomide and prednisone because of new, although sparse, ENL lesions (*23*). 

Eight patients were still receiving treatment or had just completed treatment at time of reporting. Most studies did not assess or report grade of disability. Six deaths were reported, of which 4 were attributed to DLL or sepsis secondary to DLL (*1*,*17*). One patient who died was a woman, 86 years of age, who improved after 10 days of treatment and was discharged after 2 weeks in stable condition but then died of unknown causes at home 3 months later (*16*). A man, 72 years of age, died from lung cancer after 5 months; he was described as having leprosy-like illness because, although 16S rRNA sequencing found a 100% match to *M. lepromatosis* and the patient manifested neurologic and dermatologic symptoms of LL and rhinorrhea, histopathology did not confirm mycobacteria within peripheral nerves (*18*). 

Five case-patients tested positive only for *M. leprae*: One was a woman, 43 years of age, a United States resident originally from Nepal, diagnosed with midborderline HD with possible type 1 reaction and possible LP because of an erythematous geographic skin plaque which ulcerated, but was not biopsied (*28*). Another was a woman, 37 years of age, from Colombia, HIV-positive, diagnosed with LL and LP; the LP was potentially triggered as immune reconstitution inflammatory syndrome following initiation of antiretroviral therapy (*22*). The remaining 3 patients, from the Dominican Republic, 2 with LL and 1 with BL, were from a series of 6 cases that also included 1 *M. lepromatosis*–only and 2 dual-infection cases (*31*). 

Quality assessment rated 7/12 specimen survey studies as good and 5/12 as fair quality. All but 1 study (*42*) reported details of PCR methods. Nine studies (*34*–*42*) used skin lesion or biopsy or tissue samples, 2 used both skin and lesion biopsy and skin slit smear specimens (*6*,*33*), and 1 did not explicitly state the sources of specimens (Table 2; Appendix) (*3*). Overall, surveys dated 1968–2020 reported 1,098 PCR–positive *M. lepromatosis*–only, *M. leprae*–only, or dual-infection findings from 1,428 specimens. *M. lepromatosis* was identified in 44.9% (133/296, 26 dual infection) of PCR–positive specimens from patients in Mexico or in the United States but originally from Mexico, 3.8% (5/133, 5 dual infection) of patients from Colombia, 12.5% (10/80, 3 dual infection) of patients from Brazil, and 0.9% (2/224) of patients from the Pacific-Asia region; all 157 specimens from China, 50 from Africa (Mali, Uganda), and 77 from Venezuela were positive only for *M. leprae*. For patients from Mexico, excluding those resident in the United States, *M. lepromatosis* was detected in 43.9% (116/264) of PCR–positive specimens, including 25 dual infections. For patients resident in the United States from any country of origin, *M. lepromatosis* was detected in 16.7% (20/120) of PCR–positive specimens, including 1 dual infection. 

**Table 2:** Specimen surveys investigating *Mycobacterium lepromatosis* as a cause of Hansen disease*

Ref.	Country (origin)	Specimen date range	Specimens	PCR results					HD types (remarks)
				Neg	Pos	*M. lepromatosis*	Dual infection	*M. leprae*	
(*3*)	Mexico	Not reported	64 type not reported	0	64	6	0	58	DLL 2
	Venezuela	Not reported	77 type not reported	0	77	0	0	77	
	Brazil	Not reported	33 type not reported	0	33	0	0	33	
	Mali	Not reported	48 type not reported	0	48	0	0	48	
	Others	Not reported	5 type not reported	0	5	0	0	5	
(*6*)	Mexico	Not reported	47 skin lesion biopsy	11	36	15	2	19	
	United States	2017	218 type not reported	146	72	3	0	69	
	Philippines		180 sss	0	180	0	0	180	
	United States (all but 1 born in Mexico)	1968–1994	15 skin biopsy sections	0	10	5	1	4	LL 2, DLL 4 (all originally from Mexico)
(*33*)	Colombia†	2006–2016	67 skin lesion, 25 earlobe sss	0	92	0	5	87	
(*34*)	Colombia (Cartagena, Bolívar)	2015–2020	41 skin biopsy	7	41	0	0	34	
(*35*)	Mexico	1988–2007	120 skin biopsy	33	87	55	14	18	B 12, LL 41, DLL 16
(*36*)	Brazil (Curitiba and southern Brazil)	2004–2010	52 skin biopsy	6	46	7	3	36	TT7, LL 3
	Myanmar	2007–2008	9 skin biopsy	3	6	2	0	4	LL 2
	Malaysia (19), Indonesia (3), Nepal (1), Myanmar (1)	2003–2011	31 skin biopsy	4	27	0	0	27	
	Uganda	1979–1990	4 skin biopsy	2	2	0	0	2	
(*37*)	Mexico	Current	19 skin biopsy	9	10	2	1	7	LL 2, not determined 1
(*38*)	United States (various countries of origin)‡	2011–2021	38 tissue	0	38	11	0	27	LL 11 (all originally from Mexico)
(*39*)	Mexico§	1994–2014	41 skin biopsy	12	29	8	8	13	BL 6, LL 6, DLL 4
(*40*)	Mexico	Current patients	38 skin biopsy	0	38	5	0	33	BL 1, LL 1, DLL 3
(*41*)	China	Current patients	171 skin biopsy	86	85	0	0	85	
(*42*)	China (Shandong province)	Not reported	85 skin biopsy	13	72	0	0	72	

*B, borderline (BT, BB or BL); BB, mid-borderline (borderline borderline); BL, borderline lepromatous; BT, borderline tuberculoid; DLL, diffuse lepromatous leprosy; ENL, erythema nodosum leprosum (type 2 reaction); LL, lepromatous leprosy; LP, Lucio’s phenomenon (erythema necroticans); neg., negative; pos., positive; ref., reference; sss, skin slit smear; TT, tuberculoid. †Provinces of Atlántico, Antioquia, Bolívar, Chocó, Cesar, Cundinamarca, Magdalena, Santander, Norte de Santander, Sucre, and Tolima. ‡Mexico (22), Philippines (6), Vietnam (1), India (1), Myanmar (1), Marshall Islands (2), El Salvador (2), Brazil (1), United States (2). §Yucatan (16), Guerrero (8), Michoacán (6), Guanajuato (3), Morelos (2), 1 each from Campeche, Ciudad de Mexico, Estado de Mexico, Oaxaca, Puebla, and Quintana Roo.

The distribution of HD types among 116 *M. lepromatosis*–only and 13 dual-infection patients was tuberculoid in 7 (5.4%); borderline tuberculoid, midborderline, or borderline lepromatous in 20 (15.5%); LL in 73 (56.6%); and DLL in 29 (22.5%). LP was reported in relation to 14/27 specimens from patients in Mexico or in the United States but originally from Mexico. One patient from Mexico with LL who provided a specimen positive for *M. lepromatosis* had consumed armadillo meat (*40*). 

## Discussion 

Our systematic review identified 27 case reports of HD attributed to PCR–confirmed *M. lepromatosis* infections. In addition, surveys of specimens from current patients and archived material uncovered 153 cases of *M. lepromatosis* HD. Most of those infections (60% of case reports, 87% of surveyed specimens) occurred in patients resident in Mexico or in the United States but originally from Mexico. Most (70%) of the case reports described patients with DLL, among whom half manifested LP. 

Our findings appear to substantiate the hypothesis that *M. lepromatosis* is the predominant HD pathogen in Mesoamerica and the Caribbean, and particularly in Mexico, and that it has a strong tendency to cause the DLL form of HD and, indirectly, severe LP reaction. However, there are some important caveats. First, DLL and LP were identified in Mexico in the late 19th Century by physicians Lucio Nájera and Ygnacio Alvarado and were further characterized by Fernando Latapí in 1938 (*43*,*44*). Discovery of *M. lepromatosis* in fatal cases of DLL with LP in 2 patients in the United States who were originally from Mexico (*1*), combined with the high proportion of HD cases in Mexico that were DLL, with or without LP, might have resulted in disproportionate publication of case reports and specimen surveys focused on this form of HD in this region. Laboratory expertise and resources for detecting *M. lepromatosis* are also more readily available in Mexico and the United States. However, Mexico is not an HD-endemic country, reporting an average of <200 newly detected cases per year during 2005–2021 (*45*), mostly in the states of Guerrero, Jalisco, Oaxaca, Sinaloa, and Michoacán (*46*). Although this annual average represents a relatively small number of cases, in the context of HD elimination, it is a matter of public health concern. In addition, *M. lepromatosis* has a tendency to cause severe forms including DLL, which with its nonnodular manifestation is prone to diagnosis at later stages; therefore, there remains an immense personal impact on persons affected by the disease. 

Current HD multidrug therapies appear to be effective treatments, except in the most severe cases in which patients are at risk of secondary infection. However, evidence on the apparent effectiveness of current multidrug therapy regimens in treating HD caused by *M. lepromatosis* is constrained by the small number of cases described, their clinical complexity and severity, and lack of follow-up data to characterize long-term outcomes, including permanent disabilities. 

Our review showed that *M. lepromatosis*–caused HD occurs in other countries in the Americas and, sporadically, in Asia and the Pacific. Most notably, 1 in 8 specimens from the south of Brazil were identified as *M. lepromatosis*. Brazil is an HD-endemic country with ≈20,000 newly detected cases per year. Also, the survey data in our review showed that, when type of HD was reported, a higher percentage of cases attributed to *M. lepromatosis* were LL (57%) than DLL (23%). Even a small fraction of HD cases in Brazil caused by *M. lepromatosis* would represent a large number of cases. The clear implication is that a national survey of current and newly detected HD cases in Brazil is needed, ideally using the recently validated *M. lepromatosis*/*M. leprae* diagnostic assay (*6*). Parallel studies in neighboring countries where *M. lepromatosis* and DLL are perhaps more prevalent, would yield sequence data that could be used to investigate the distribution of *M. lepromatosis* variants and lineages, including drug-resistant strains, to achieve the same level of understanding as for *M. leprae* (*5*,*47*). Whether *M. lepromatosis* has a pathogenic tendency toward causing DLL and whether certain population groups are more susceptible to developing this form of HD can be investigated by pathogen and host genomic testing across the HD spectrum and in different populations. From a One Health perspective, we know that zoonotic transmission of *M. leprae* presents a risk to human health (*48*), and *M. lepromatosis* has been detected in red squirrels (*Sciurus vulgaris*) from the British Isles, including Ireland (*49*). Given that 4 case-patients with HD caused by *M. lepromatosis* in our review had direct or indirect contact with wild armadillos, a survey of archived specimens or specimens from freshly caught armadillos in Mexico and Brazil is warranted (*50*). 

The narrowly focused scope, sensitivity, and specificity of lepromatosis as a search term and the relatively few references included in our review give us confidence that all relevant studies were identified. Quality of reporting was good in 61% of included studies and fair in the remainder. The tendency of studies to focus on DLL in Mesoamerica, possibly resulting in observational and publication biases for case reports and sampling bias for surveys, were the main sources of bias in our review, although there were several large studies from other regions with null findings for *M. lepromatosis*. A key quality item considered for this review was adequate description of PCR methods, which most, but not all, studies provided. Specimen surveys more consistently described PCR methods, including targets and primers, than did case reports, some of which covered time periods during which those methods were still being developed. *M. lepromatosis* does not manifest only as DLL, but most specimen surveys did not provide clinical data for the patients sampled. Even when HD type was stated, misclassification was possible unless HD specialists or reference centers were involved in diagnosis. Although we cannot entirely preclude the possibility of double counting, we identified only a few cases that were reported twice and contacted the authors of 2 studies when geography and time span suggested that possibility to confirm that there was no overlap (*33*,*34*).

It is perhaps remarkable that a new species causing an endemic disease of major public health impact has not prompted larger-scale studies to determine its true prevalence. Even if options for patient management are determined by clinical manifestations of HD rather than its etiologic agents, understanding disease attribution and distribution of a highly pathogenic species are clearly important, and the availability of validated PCR methods enables large-scale epidemiologic studies to be conducted. 

In conclusion, clinicians need to be aware that Hansen disease of various forms can be caused by either *M. leprae* or *M. lepromatosis*. Current multidrug therapy regimens appear to be effective regardless of infecting species. 

Appendix Additional information about a literature review of *Mycobacterium lepromatosis* as a cause of Hansen disease.
